# Retraction: On soft expert topological spaces

**DOI:** 10.1186/2193-1801-1-42

**Published:** 2012-10-24

**Authors:** Sabir Hussain, Mohammad Ahmad Alghamdi

**Affiliations:** 1 Department of Mathematics, Yanbu University, P. O. Box 31387, Yanbu Alsinaiyah, Saudi Arabia; 2 Department of Mathematics, King Abdul Aziz University, P. O. Box 80203, Jeddah, 21589 Saudi Arabia

After publication of article [1], we became aware of the fact that sections of this article were taken verbatim without quotation from [2] and [3] listed below. In addition, ‘example 2’ and ‘algorithm 4.1’ as presented in this article are identical to ‘example 3.3’ from [3] and ‘algorithm 4.1’ from [2]. In light of these problems and in consultation with the journal's Section Editor for Mathematics we have decided to retract this article from SpringerPlus.
